# S05-1: Dutch national fall prevention program - implementation challenges

**DOI:** 10.1093/eurpub/ckae114.218

**Published:** 2024-09-26

**Authors:** Liesbeth Preller

**Affiliations:** Dutch Knowledge Centre for Sport & Physical Activity

## Abstract

**Purpose:**

In the Netherlands, an ambitious national multi-domain fall prevention program for older people is being implemented starting in 2023 as part of a broader prevention plan. This is done because of the high burden of falls for healthcare capacity and costs. Large responsibility is given to municipalities for implementation at the local level, and to regional public health services at a higher level.

**Project description:**

The program is developed based on existing evidence and by consulting a wide range of stakeholders. This led to a chain approach including description of screening, fall prevention physical activity programs (FPPs), Physical Activities (PA) outside these FPPs for people with no or limited fall risk and as follow up after participation to FPPs. Each year 3% of people over 65 should attend an FPP VeiligheidNL is responsible for coordinating development and subsequent implementation.

Local implementation is generally done or under supervision of the local policy department of Sport. This is supported by involving several national and regional stakeholders on public health and local policy. Additionally, national and regional sports organisations fill gaps experienced by municipalities and other stakeholders.

Preliminary evaluation shows that local implementation largely varies depending on the role taken by municipalities and local sports and healthcare organisations, and by regional health service organisations.

Dissemination to all municipalities is guaranteed because of designated funding in combination with obligations for health insurance companies to reimburse the costs of participation to FPP for the (estimated 1% of 65) people with the highest fall risk.

**Conclusions:**

The national program leads to broader knowledge about and attention to PA for older people, development of supportive tools and a slowly increasing number of people attending FPP. Still many challenges have to be overcome, such as reach of the target group, local intersectoral collaboration, and scaling up to obtain sufficient qualified trainers for FPP and sufficient structural PA at local sports and PA providers.

